# Supplementary material for: Analysis of the light intensity dependence of the growth of Synechocystis and of the light distribution in a photobioreactor energized by 635 nm light
Source: PeerJ. 2018 Jul 27;6:e5256. doi: 10.7717/peerj.5256 (PMC6065478; doi:10.7717/peerj.5256)
Supplement: Supplemental Information 1 — This file reports additional figures displaying slice trend of dispersed phase, photobioreactor characteristics, full set of equations employed in the modelling approach and additional physiological features for Synechocystis. [file peerj-06-5256-s001.docx]

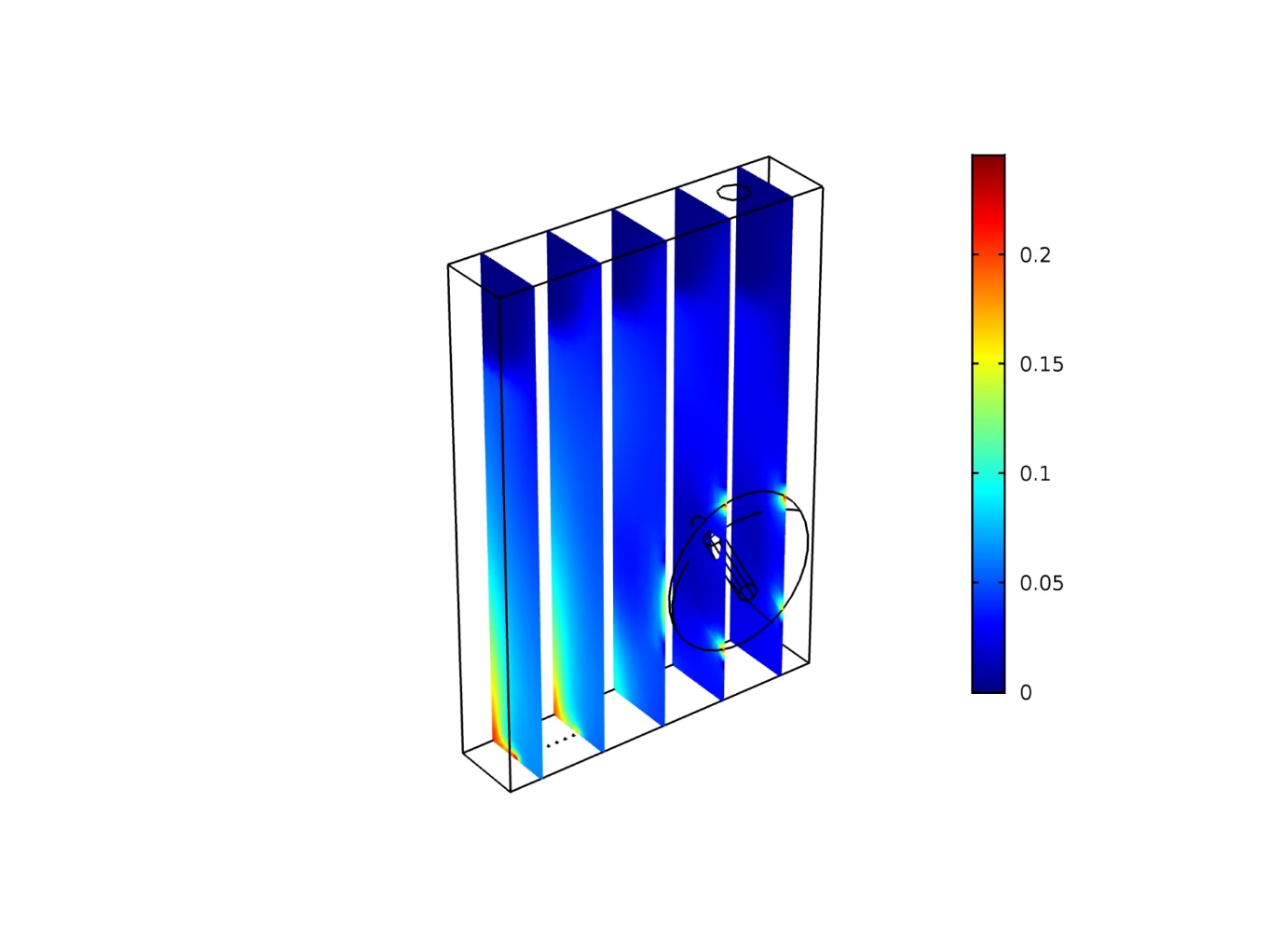


**Figure S13D slice trend of dispersed phase.**

The dispersed phase mixture of CO_2_ and N_2_ is plotted at 24 h and for incident light intensitiy, *I_inc_*, equal to 300 µmol photons m^-2^ s^-^1


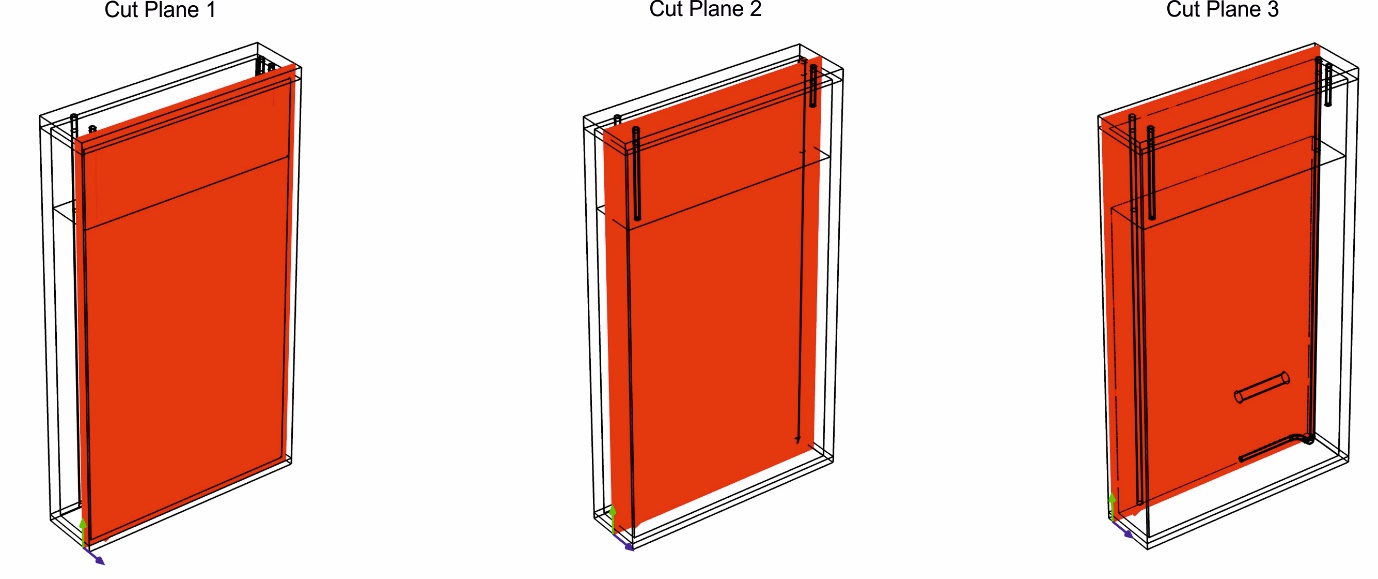


**Figure S2**

| **Equation** | **Domain** | **Mathematical model** |
| --- | --- | --- |
| $\frac{\partial C_{i}}{\partial t}+\nabla\cdot C_{i}\mathbf{u}+\nabla\cdot\mathbf{J}_{i}^{*}=R_{i}$ | 3-4-6-7 | MASS TRANSFER |
| $\mathbf{N}_{i}=\mathbf{J}_{i}^{*}+C_{i}\mathbf{u}$ | 3-4-6-7 |  |
| $\mathbf{J}_{i}^{*}=-D_{i}\nabla C_{i}$ | 3-4-6-7 |  |
| $\frac{\partial\rho}{\partial t}+\nabla\cdot(\rho\mathbf{u})=0$ | 3-4-6-7 | FLUID-DYNAMICS |
| $\frac{\partial(\rho\mathbf{u})}{\partial t}+\nabla\cdot(\rho\mathbf{uu})=-\nabla p-\nabla\cdot\mathbf{\tau}+\rho\mathbf{g}+\mathbf{F}$ | 3-4-6-7 |  |
| $\mathbf{\tau}=-\mu[(\nabla\mathbf{u}+(\nabla\mathbf{u})^{\text{T}})-\frac{2}{3}(\nabla\cdot\mathbf{u})\mathbf{I}]$ | 4-6-7 |  |
| $\rho\frac{\partial k}{\partial t}+\rho\mathbf{u}\cdot\nabla k=\nabla\cdot((\mu+\frac{\mu_{\text{T}}}{\sigma_{k}})\nabla k)+\mathbf{P}_{k}-\rho\varepsilon$ | 4-6-7 |  |
| $\rho\frac{\partial\varepsilon}{\partial t}+\rho\mathbf{u}\cdot\nabla\varepsilon=\nabla\cdot((\mu+\frac{\mu_{\text{T}}}{\sigma_{\varepsilon}})\nabla\varepsilon)+C_{\varepsilon1}\frac{\varepsilon}{k}\mathbf{P}_{k}-C_{\varepsilon2}\rho\frac{\varepsilon^{2}}{k}$ | 4-6-7 |  |
| $\mathbf{P}_{k}=\mu_{\text{T}}(\nabla\mathbf{u}:(\nabla\mathbf{u}+(\nabla\mathbf{u})^{\text{T}})-\frac{2}{3}(\nabla\cdot\mathbf{u})^{2})-\frac{2}{3}\rho k\nabla\cdot\mathbf{u}$ | 4-6-7 |  |
| $\mu_{\text{T}}=\rho C_{\mu}\frac{k^{2}}{\varepsilon}$ | 4-6-7 |  |
| $\mathbf{\tau}=-(\mu+\mu_{\text{T}})[(\nabla\mathbf{u}+(\nabla\mathbf{u})^{\text{T}})-\frac{2}{3}(\nabla\cdot\mathbf{u})\mathbf{I}]+\frac{2}{3}\rho k\mathbf{I}$ | 4-6-7 |  |
| $\emptyset_{c}+ \emptyset_{d}=1$ | 3-4-6-7 |  |
|  | 3-4-6-7 |  |
|  | 3-4-6-7 |  |
| $\mu_{eff}=\mu_{i}+\mu_{i,T}$ | 3-4-6-7 |  |
|  | 3-4-6-7 |  |
|  | 3-4-6-7 |  |
|  | 3-4-6-7 |  |
| $D_{md}=\frac{\mu_{\text{T}}}{\rho\sigma_{\text{T}}}$ | 3-4-6-7 |  |
|  | 3-4-6-7 |  |
|  | 3-4-6-7 |  |
| $\rho C_{p}\frac{\partial T}{\partial t}+ \rho C_{p}\mathbf{u}\cdot\nabla T+\nabla\cdot\mathbf{q}=Q+Q_{r}$ | all | HEAT TRANSFER WITH RADIATION |
| $\mathbf{q}=-k_{c}\nabla T$ | all |  |
| $Q_{r}=\kappa(G-4\pi I_{b})$ | 3-4-6-7-8 |  |
| $\nabla\cdot\left( D_{P1}\nabla G \right)+\kappa\left( G-4\pi I_{b} \right)=0$ | 3-4-6-7-8 |  |
| $G=\int_{4\pi} I\left( \Omega\right)d\Omega$ | 3-4-6-7-8 |  |
| $D_{P1}=\frac{1}{3\kappa+\sigma_{S}(3-a_{1})}$ | 3-4-6-7-8 |  |
| $\Omega\cdot\nabla I\left( \Omega\right)=\kappa I_{b}\left( T \right)-\beta I\left( \Omega\right)+\frac{\sigma_{S}}{4\pi}\int_{4\pi} I\left( \Omega^{'} \right)\Phi\left( \Omega^{'},\Omega\right)d\Omega'$ | 3-4-6-7-8 |  |
| $\beta=\kappa$ +$\sigma_{S}$ | 3-4-6-7-8 |  |
| $I_{b}\left( T \right)=\frac{n_{r}^{2}\sigma T^{4}}{\pi}$ | 3-4-6-7-8 |  |
| $\frac{I}{I_{0}}=exp(-\beta z)$ | 3-4-6-7-8 |  |
| $\frac{I}{I_{0}}=exp\left( -\frac{3{}_{d}z}{d_{b}}-K_{a}z \right)$ | 3-4-6-7-8 |  |
| $\mu_{gr}=\mu_{max}\frac{C}{k_{s}+C}$ | 6-7 | GROWTH KINETICS |
| $\mu_{gr}=\frac{\mu_{max}\cdot I}{I+k_{s}+\frac{I^{2}}{k_{i}}}$ | 6-7 |  |
|  |  |  |

**Table S1**

| **Light intensity**  **(µmol photons m^-2^ s^-1^)** | | **OD_680_/OD_720_** | |
| --- | --- | --- | --- |
|  |  | |  |
| 50 | 1.774 ± 0.002 | |  |
| 200 | 1.548 ± 0.042 | |  |
| 300 | 1.518 ± 0.009 | |  |
| 500 | 1.496 ± 0.022 | |  |
| 800 | 1.475 ± 0.010 | |  |
| 950 | 1.545 ± 0.035 | |  |
| 1460 | 1.597 ± 0.041 | |  |
| 200* | 1.557 ± 0.017 | |  |
|  |  | |  |
|  |  |  |  |

**Table S2**

| Domain | Element size | [m] |
| --- | --- | --- |
| Reator | Height | 0.1983 |
|  | Width | 0.11 |
|  | Thickness | 0.024 |
| Sparger | Diameter of inlet | 0.002 |
|  | Diameter of holes | 0.0004 |
|  | Number of holes | 7 |
|  | Lenght | 0.03 |
| Anchor | Diameter | 0.006 |
|  | Lenght | 0.035 |
| Vessel | Thickness | 0.0033 |

**Table S3**

|  | Gas phase | Liquid phase |
| --- | --- | --- |
| Inlet velocity | 150 ml/min | 0 |
| Outlet pressure | Gas outlet condition | Patm, no out |
| Gravity force | g=(9.81)ms^-2^ g=(9.81)ms^-2^ | |
| Wall conditions | No gas flux at the reactor boundary;  sparger non-slip model | Wall functions |
| Incident light intensity | 50/200/300/500/800/950/1460 µmol photons m^-2^s^-1^ | |

**Table S4**

| Iin | Iout | Iads | Iads | Vol | mu |  |  | efficiency |
| --- | --- | --- | --- | --- | --- | --- | --- | --- |
| mmol photons m-2s-1 | mmol photons m-2s-1 | mmol photons m-2s-1 | mol m-3h-1 | L | h-1 | gDW L-1 | mol photons g DW-1 | gDW mol photons-1 |
| 50 | 31.75 | 18.25 | 2.74 | 0.38 | 0.05 | 0.15 | 0.37 | 2.70 |
| 200 | 140.20 | 59.80 | 8.97 | 0.38 | 0.11 | 0.14 | 0.61 | 1.64 |
| 300 | 219.00 | 81.00 | 12.15 | 0.38 | 0.12 | 0.15 | 0.75 | 1.33 |
| 500 | 386.40 | 113.60 | 17.04 | 0.38 | 0.11 | 0.15 | 1.11 | 0.90 |
| 800 | 631.55 | 168.45 | 25.27 | 0.38 | 0.10 | 0.15 | 1.68 | 0.60 |
| 950 | 770.45 | 179.55 | 26.94 | 0.38 | 0.09 | 0.15 | 2.11 | 0.48 |

**Table S5**
